# Novel Exploratory Transcriptomic Candidates as Biomarkers and Cancer Hallmark Fingerprints for Ovarian Endometroid and Clear Cell Carcinomas in Women

**DOI:** 10.3390/antiox15080979

**Published:** 2026-08-06

**Authors:** Pawel Kordowitzki, Kejun Ying

**Affiliations:** 1 Department of Basic and Preclinical Sciences, Nicolaus Copernicus University, 87-100 Torun, Poland; 2 Department of Gynecology, Campus Virchow Klinikum, Charité-Universitätsmedizin Berlin, Augustenburger Platz 1, 13353 Berlin, Germany; 3 Institute of Advanced Studies, Nicolaus Copernicus University, 87-100 Torun, Poland; 4 Tse-hsi (T. H.) Chan School of Public Health, Harvard University, Boston, MA 02115, USA; keying@stanford.edu; 5 Wu Tsai Neurosciences Institute, Stanford University, Stanford, CA 94305, USA

**Keywords:** *RPS28*, *EPAS1*, *ALKBH2*, *KRAS*, *DCLRE1A*, *LRRK2*, consensus biomarkers, cancer hallmarks, ovarian endometroid cancer, ovarian clear cell ovarian cancer, endometriosis-associated ovarian cancer, women, hypoxia, oxidative stress

## Abstract

Background: Endometriosis-associated ovarian cancers (EAOCs), encompassing clear cell (CC) and endometrioid carcinomas (EC), constitute distinct biological entities yet lack robust biomarkers for precise classification, prognostication, and therapeutic decision-making in women. Therefore, we aimed to describe novel biomarkers. Methods: In this study, we conducted an integrated transcriptomic analysis, powered by machine learning, to discover novel consensus biomarkers and delineate cancer hallmark signatures specific to EC and CC. Drawing on gene expression profiles from EAOC specimens, we merged differential expression analysis with LASSO regression and Random Forest classification to generate a reliable biomarker panel that effectively distinguishes EC from CC. Kaplan–Meier survival analyses and mutation analyses have been performed for selected biomarker genes. Results: Novel biomarkers, among others, the genes *RPS28*, *EPAS1*, *ALKBH2*, and *DCLRE1A*, uncover extensive transcriptional alterations tied to hypoxia signaling, oxidative stress, DNA repair, and metabolic reprogramming. Gene Ontology and pathway enrichment analyses revealed synchronized upregulation of epithelial–mesenchymal transition, TNF-α/NF-κB signaling, oxidative stress, hypoxia, and KRAS signaling pathways. Conclusions: Our work establishes novel exploratory transcriptomic candidates for innovative consensus biomarkers, yielding novel diagnostic and prognostic insights into EAOC and supporting further study of subtype-associated expression programs. The current study was designed primarily as an integrative computational investigation aimed at identifying candidate genes and molecular pathways distinguishing CC from EC.

## 1. Introduction

Ovarian cancer represents a significant global health challenge, ranking as the eighth most common cause of cancer and cancer-related mortality in women worldwide [1]. Over the past decade, ovarian cancer mortality has exhibited steeper declines relative to incidence rates, likely attributable to modest improvements in survival among patients with advanced disease alongside a trend toward earlier-stage diagnosis [2]. While high-grade serous ovarian carcinomas account for the majority of epithelial ovarian malignancies [3], a distinct subset, known as endometriosis-associated ovarian cancer (EAOC), arises from the malignant transformation of endometriotic lesions [4,5,6]. The association between endometriosis and ovarian cancer is predominantly observed in endometrioid carcinoma (EC) and clear cell carcinoma (CC), which represent distinct histological subtypes of epithelial ovarian cancer [7,8,9,10,11]. Epidemiological studies have demonstrated a significantly increased risk of developing ovarian CC (3.4-fold) and ovarian EC (2.3-fold) in women with endometriosis [12]. These cancers, often classified as Type I ovarian carcinomas, typically exhibit relatively stable genomes and are thought to evolve from visible precursor lesions, such as atypical endometriosis [9,12,13]. Atypical endometriosis, characterized by cytological atypia within endometriotic glands, is considered a true precursor to EAOC and is found in a substantial proportion of ovarian cancers arising from endometriosis [5,14,15,16]. Ovarian clear cell carcinoma, for instance, is histopathologically defined by clear and “hobnail” cells and is known for its genetic stability and often suboptimal response to conventional platinum-based chemotherapy [7,17,18].

Beyond specific genetic mutations, the oxidative stress exposure in the microenvironment of endometriotic lesions contributes significantly to malignant progression. Endometriosis is characterized by chronic inflammation, oxidative stress, and the production of reactive oxygen species (ROS) [19,20,21,22]. This persistently high ROS level and inflammatory milieu foster genomic instability, providing fertile ground for the accumulation of additional genetic alterations, which can ultimately lead to malignant transformation [23,24]. The multiple molecular traits shared between endometriosis and invasive cancer, such as inflammation, tissue invasion, angiogenesis, ROS-related damage, and enhanced local estrogen production, emphasize the intrinsic predisposition for malignant change within these lesions [10,25].

Oxidative stress is increasingly recognized as a biologically relevant feature of endometriosis-associated ovarian cancer, particularly ovarian clear cell carcinoma [26,27,28,29]. Alterations in antioxidant systems, including superoxide dismutases (SOD1 and SOD2) and the NRF2 (NFE2L2)–KEAP1 pathway, have been implicated in maintaining redox homeostasis and supporting tumor-cell survival under oxidative stress [30,31]. In parallel, increased activity of NADPH oxidase (NOX) family enzymes, which constitute important sources of intracellular ROS, may contribute to persistent oxidative signaling and genomic instability [32]. Collectively, these alterations suggest that dysregulated ROS production and antioxidant defense constitute an interconnected molecular signature of endometriosis-associated carcinogenesis, although the relative contributions of individual oxidative stress pathways may differ between CC and EC. Further characterization of these redox-associated signatures may therefore help clarify the molecular divergence of EAOC subtypes and identify potential biomarkers and therapeutic vulnerabilities.

Despite increased understanding of the molecular and ROS-related landscape of EAOC, challenges persist in early diagnosis and patient management [33,34]. The precise mechanisms and molecular events involved in the shift from benign endometriosis to atypical lesions and ultimately to invasive cancer require further elucidation [9]. The hallmarks of cancer describe the fundamental capabilities acquired by cancer cells, providing a framework for understanding the complexities of ovarian cancer development and progression [35,36]. Early biomarkers are vital for improving ovarian cancer prognosis, as most cases are diagnosed at advanced stages, leading to poor survival rates [37,38,39]. Despite efforts, current screening methods, like CA125, lack the necessary sensitivity and specificity for effective early detection [40,41].

Therefore, in our sophisticated, integrative transcriptomic and machine learning-driven analyses, we aim to identify novel consensus biomarkers and cancer hallmark fingerprint genes in ovarian endometroid and clear cell carcinomas, potentially transforming the diagnostic landscape for these two histological subtypes and providing a better understanding of the relevance of oxidative stress-related genes in endometriosis-associated ovarian cancer. The aim of our work is to establish novel exploratory transcriptomic candidates for innovative consensus biomarkers, yielding novel diagnostic and prognostic insights into EAOC and supporting further study of subtype-associated expression programs. The current study was designed primarily as an integrative computational investigation aimed at identifying candidate genes and molecular pathways distinguishing CC from EC.

## 2. Materials and Methods

### 2.1. Data Acquisition and Preprocessing

Gene expression data were obtained from the NCBI Gene Expression Omnibus (GEO) dataset GSE73614, profiled on the Agilent-014850 (Agilent Technologies, Santa Clara, CA, USA) Whole Human Genome Microarray 4x44K platform (GPL6480). The dataset contains endometriosis-associated ovarian cancer (EAOC) samples comprising clear cell carcinoma (*n* = 24) and endometrioid carcinoma (*n* = 35). Probe-level data were mapped to gene symbols using platform annotations, with multiple probes per gene aggregated by mean expression. Genes with missing values were imputed using the gene-wise median, and low-variance genes (variance < 0.01) were filtered, yielding 14,381 genes for downstream analysis. The 0.01 variance cutoff was used as a pragmatic dimensionality-reduction setting and reproduced the supplied 14,381-gene matrix. The available GSE73614 records include histotype and array identifiers. Complete sample-level covariates for age, menopausal status, stage, grade, treatment history, outcome, and batch were unavailable for confounder adjustment. All patients received platinum-based postoperative chemotherapy.

### 2.2. Differential Expression Analysis

Differentially expressed genes (DEGs) between clear cell and endometrioid carcinoma were identified using Welch’s t-test. *p*-values were adjusted for multiple testing using the Benjamini–Hochberg method to control the false discovery rate (FDR). Genes with adjusted *p* < 0.05 and |log2 fold change| ≥ 0.3 were considered significant in the primary discovery analysis. Log2 fold change was defined as mean clear cell minus mean endometrioid expression. Sensitivity analyses repeated the same test and FDR procedure at absolute log2 fold-change thresholds of 0.5 and 1.0. We recorded DEG counts and panel-gene retention at each threshold. Set nesting across thresholds was also examined.

### 2.3. Machine Learning Feature Selection and Classification

LASSO Logistic Regression: L1-penalized logistic regression was performed using 5-fold stratified cross-validation to select the optimal regularization parameter. Features with non-zero coefficients were retained for downstream analysis. Random Forest Classification: A Random Forest classifier (500 trees, max depth = 10, balanced class weights) was trained on LASSO-selected features. Model performance was evaluated using 5-fold stratified cross-validation, with metrics including accuracy, AUC-ROC, precision, recall, and F1-score. Feature importance was calculated using Gini impurity. Consensus Biomarkers: Genes appearing in both the top 100 LASSO-selected features (by absolute coefficient) and top 100 Random Forest features (by importance) were designated as consensus biomarkers. For the revised internal performance analysis, endometrioid carcinoma was coded as class 1 and clear cell carcinoma as class 0. We used repeated stratified 5-fold outer cross-validation with five repeats. The outer splits used seed 20260714; inner cross-validation and model fitting used 20260714 plus the outer-fold index. Within each outer training set, missing values were median-imputed, genes with training-sample variance below 0.01 were removed, and retained features were standardized. L1-penalized logistic regression used the liblinear solver with balanced class weights. The iteration limit was 5000. The regularization grid was expressed as scikit-learn C values and selected by 4-fold cross-validation using ROC-AUC from 0.03, 0.1, 0.3, 1, 3, 10, 30, and 100. Random Forest hyperparameters were then selected by 4-fold cross-validation using ROC-AUC. The grid used 500 trees, balanced class weights, sqrt feature sampling, maximum depth 6 or unrestricted, minimum split 2 or 5, and minimum leaf 1 or 2. Outer-test probabilities were classified at 0.5.

Variance-threshold sensitivity was assessed separately at 0, 0.001, 0.005, 0.01, 0.02, and 0.05 using repeated stratified 5-fold cross-validation with three repeats. The outer splits used seed 20265714; inner cross-validation and model fitting used 20265714 plus the fold index. L1 C was reselected within each outer training partition. To isolate the variance threshold, the Random Forest used 500 trees, maximum depth 10, minimum split 5, minimum leaf 2, sqrt feature sampling, balanced class weights, and a 0.5 decision threshold. Imputation, variance filtering, and scaling were fitted within outer training data.

For an exploratory cross-cohort assessment, we trained a separate rank-based clear cell-versus-endometrioid classifier after exact feature matching between GSE73614 and GSE226870. Model fitting and selection of the 29-gene feature set used GSE73614 labels only; the model parameters and 0.5 threshold were fixed before evaluation without refitting in GSE226870. This analysis did not evaluate or validate the submitted 37-gene panel.

### 2.4. Pathway Enrichment Analysis

Gene set enrichment analysis was performed on significant DEGs using Enrichr against MSigDB Hallmark gene sets and GO Molecular Function, GO Cellular Component, and Reactome pathways. Pathways with adjusted *p* < 0.05 were considered significantly enriched.

### 2.5. Cancer Hallmark Genes and Kaplan–Meier Survival Analysis

Cancer hallmark genes have been analyzed by merging 6763 genes from available mapping resources on CancerHallmarks.com, as recently published by Menyhart et. al. (2025) [42]. The gene-specific Kaplan–Meier analysis was performed using the online plotter tool established by Gyorffy (2023) [43].

### 2.6. Identification of Mutations Influencing the Expression of Selected Target Genes

The muTarget cancer biomarker tool has been used to analyze a target gene and identify mutations that affect its expression. The selected target gene could serve as a biomarker to select patients who may benefit from personalized targeted therapy. The analysis was run as previously described and established by Nagy and Gyorffy (2021) [44]. Briefly, muTarget uses paired somatic mutation and RNA-sequencing data from TCGA to examine the mutation status of genes across the available tumor cohort. For each candidate mutated gene, patients are divided into two groups: patients with a mutation in that gene and patients without a mutation in that gene (wild-type). The expression of the selected target gene (*KRAS* and *EPAS1*) is compared between these two groups. The statistical significance of the difference is assessed using the Mann–Whitney U test, while the magnitude of the difference is evaluated using the fold change between the mutant and wild-type groups. Genes meeting the predefined significance criteria are retained and ranked according to the strength of their statistical association. The original muTarget methodology used *p* < 0.01 and a fold-change threshold corresponding to approximately >1.4 or <0.714.

### 2.7. Statistical Analysis

All statistical analyses were performed in Python 3 using scikit-learn (v1.x), scipy, statsmodels, and gseapy. Visualizations were generated using matplotlib and seaborn with colorblind-friendly palettes. *p*-values < 0.05 were considered statistically significant unless otherwise specified. The revised classifier analyses used Python 3.14.5, NumPy 2.4.5, pandas 2.3.3, SciPy 1.16.3, and scikit-learn 1.8.0.

## 3. Results

### 3.1. Differential Gene Expression and Consensus Biomarkers in Ovarian CC and EC

**Figure 1:**
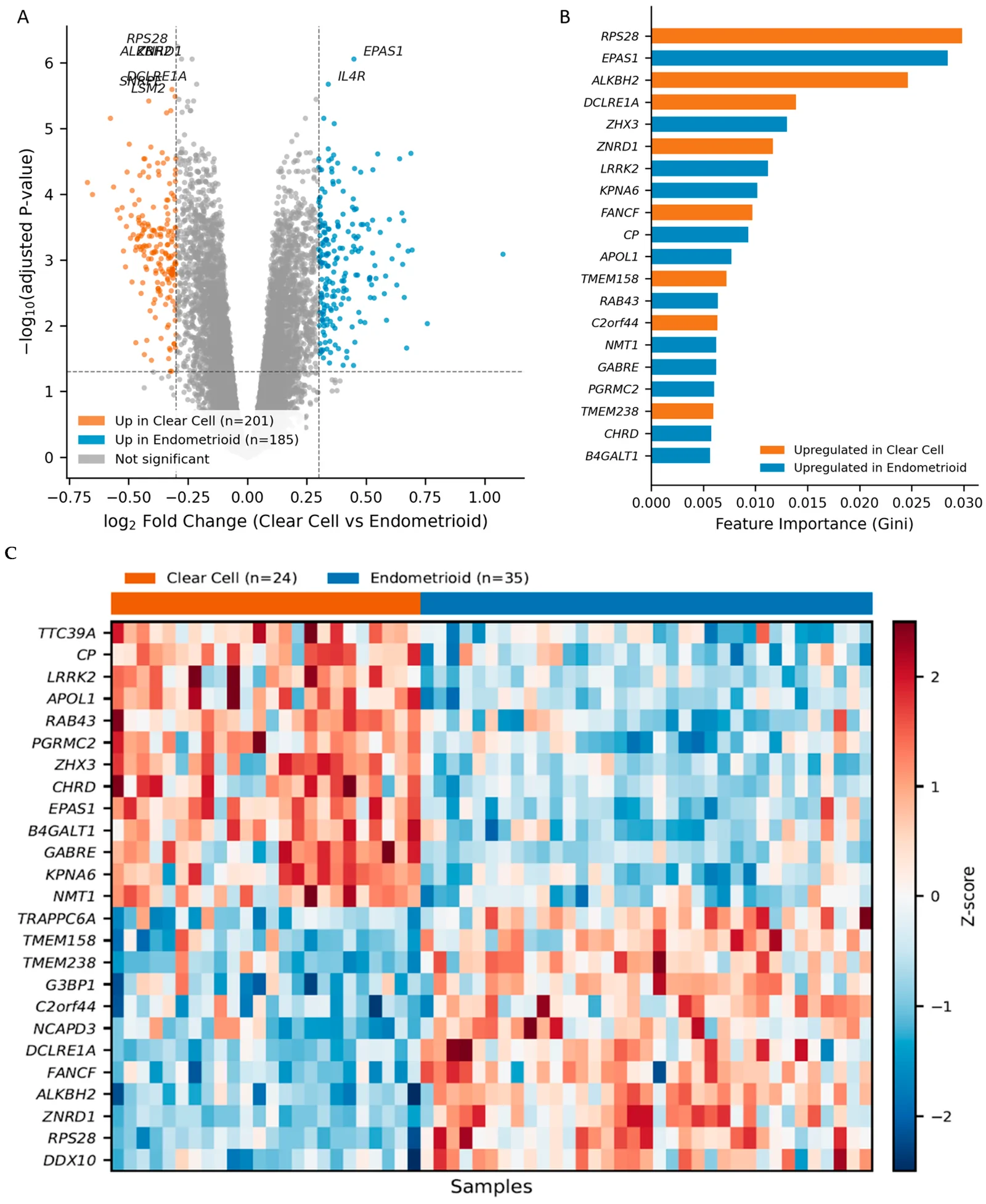
Differential gene expression between clear cell and endometrioid ovarian carcinoma. Panel (**A**) shows a Volcano plot of 14,381 genes from GSE73614. Orange: genes upregulated in clear cell carcinoma; blue: genes upregulated in endometrioid carcinoma; gray: not significant. Dashed lines indicate significance thresholds (adjusted *p* < 0.05, |log2FC| > 0.3). Top differentially expressed genes are labeled. Panel (**B**) shows the top 20 consensus biomarker genes ranked by Random Forest feature importance. Genes were selected by both LASSO and Random Forest (top 100 overlap). Orange: upregulated in clear cell carcinoma; blue: upregulated in endometrioid carcinoma. Feature importance was calculated using Gini impurity. Panel (**C**) shows the expression heatmap of 25 consensus biomarker genes. Here, Z-score-normalized expression across 59 EAOC samples is shown for 25 consensus biomarkers, annotated on the left. Samples are grouped by histological subtype (clear cell, *n* = 24; endometrioid, *n* = 35). Genes are hierarchically clustered, and the color bar indicates the sample subtype.

**Figure 2:**
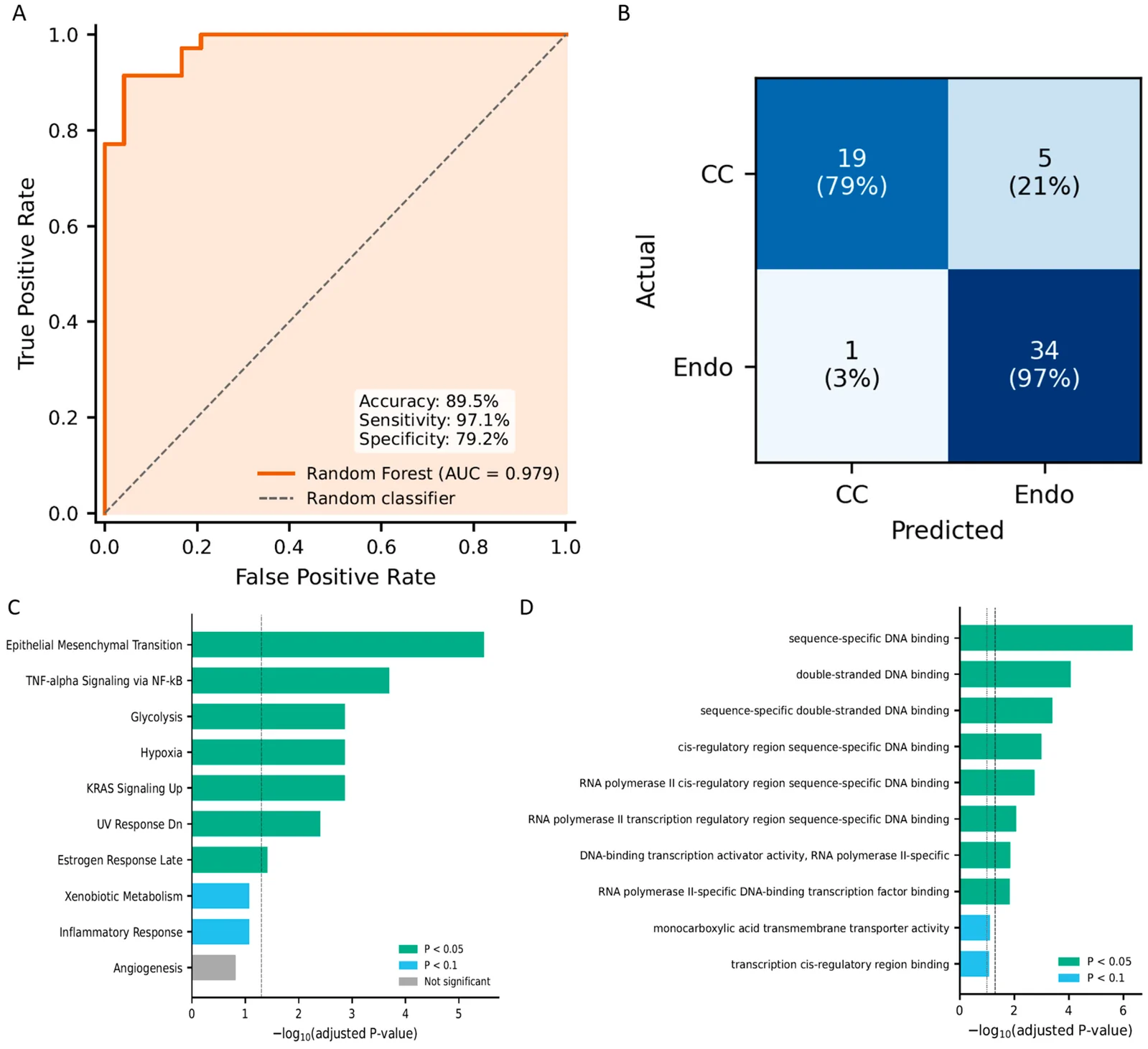
Random Forest classifier performance for EAOC subtype prediction and hallmark pathway enrichment analysis. Panel (**A**) depicts the receiver operating characteristic (ROC) curve from a 5-fold cross-validation, whereas Panel (**B**) depicts the confusion matrix from 5-fold cross-validation. Panel (**C**) shows the enrichment of 386 significant DEGs against MSigDB Hallmark gene sets. Green: adjusted *p* < 0.05; light blue: adjusted *p* < 0.1; gray: not significant. Dashed line indicates *p* = 0.05 threshold. Panel (**D**) shows the Gene Ontology Molecular Function enrichment analysis. Here, the enrichment of 386 significant DEGs against GO Molecular Function terms is depicted. Green: adjusted *p* < 0.05; light blue: adjusted *p* < 0.1. Dashed lines indicate *p* = 0.05 and *p* = 0.1 thresholds.

Differently expressed genes (Figure 1A) appearing in both the top 100 LASSO-selected features (by absolute coefficient) and top 100 Random Forest features (by importance) were designated as consensus biomarkers. According to the feature importance (Gini) analysis, the genes *RPS28*, *ALKBH2*, *DCLRE1A*, and *ZNRD1* were highly significantly upregulated in CC samples, whereas *EPAS1*, *ZHX3*, *LRRK2*, and *KPNA6* were among the most significantly upregulated in EC samples, which demonstrates a robust transcriptional reprogramming distinguishing CC from EC (Figure 1). Besides the latter-mentioned genes, the following were also detected among the top 20 consensus biomarkers: *B4GALT1*, *CHRD*, *TMEM238*, *PGRMC2*, *GABRE*, *NMT1*, *C2orf44*, *RAB43*, *TMEM158*, *APOL1*, *CP*, and *FANCF* (Figure 1B). In the heatmap of Z-score-normalized expression values for the top classifier genes across all samples, a clear clustering of samples based on histological subtype is evident, with distinct expression patterns separating CC from EC (Figure 1C). Genes such as EPAS1 and RPS28 exhibit consistent subtype-specific expression, reinforcing their potential as diagnostic biomarkers. Furthermore, the gene-based classifier’s performance in discriminating CC from EC samples was assessed using a receiver operating characteristic (ROC) curve. In the separate variance-threshold sensitivity analysis, mean balanced accuracy across thresholds 0, 0.001, 0.005, 0.01, 0.02, and 0.05 ranged from 0.793 to 0.830, and mean ROC-AUC ranged from 0.864 to 0.911. At the 0.01 threshold, the corresponding values were 0.799 and 0.870. Performance did not show a sharp transition at 0.01. These values are compared only within the sensitivity design. The high AUC value underscores the strong discriminative power of the selected gene signatures (Figure 2A). Furthermore, the confusion matrix of classification performance, as presented in Figure 2B, summarizes the classification accuracy between CC and EC. In this analysis, 79% of the CC samples were correctly classified, while 21% were misclassified as EC. Conversely, 97% of EC samples were correctly predicted, with only 3% misclassified as CC. These results indicate particularly high robustness of the model in identifying EC, with slightly lower but still strong performance for CC. The lower classification performance observed for CC may reflect a combination of factors, including class imbalance and genuine biological overlap between endometrioid and clear cell ovarian carcinomas. Using the primary 14,381-gene matrix and FDR below 0.05, 386, 44, and 1 genes met absolute log2 fold-change thresholds of 0.3, 0.5, and 1.0, respectively. The 44-gene and 1-gene sets were nested within the 0.3 set. Of the submitted 37-gene panel, 12, 2, and 0 genes met both the FDR and corresponding fold-change criteria. The 0.3 cutoff was retained for exploratory discovery. The panel is described as exploratory because retention fell to 2 and 0 genes at the stricter cutoffs.

### 3.2. Pathway Enrichment Analysis and Gene Ontology Molecular Function

The pathway enrichment analysis identified multiple significantly enriched hallmark pathways in clear cell and endometroid ovarian carcinoma (Figure 2C). The most strongly enriched signatures were epithelial–mesenchymal transition (EMT), TNF-α signaling via NF-κB, glycolysis, and hypoxia. Additional significantly enriched pathways included KRAS signaling (upregulated), UV response, and late estrogen response. Pathways related to xenobiotic metabolism, inflammatory response, and angiogenesis showed weaker enrichment or borderline statistical significance. The Gene Ontology Molecular Function analysis demonstrated significant enrichment of categories related to sequence-specific DNA binding and transcriptional regulation (Figure 2D). The top enriched terms included sequence-specific DNA binding, double-stranded DNA binding, cis-regulatory region DNA binding, and RNA polymerase II transcription regulatory region sequence-specific DNA binding. In addition, enrichment was observed for RNA polymerase II-specific transcription factor activity and transcription factor binding, indicating broad involvement of transcriptional control mechanisms in endometriosis-associated ovarian cancer. A metabolic component was also reflected by enrichment of monocarboxylic acid transmembrane transporter activity. In particular, the enrichment of EMT-related pathways, the upregulation of KRAS signaling, and transcriptional regulatory functions reflect a characteristic molecular profile of endometrioid and clear cell ovarian carcinoma.

### 3.3. Cancer Hallmark-Based Functional Landscape of Top Biomarker Genes

**Figure 3:**
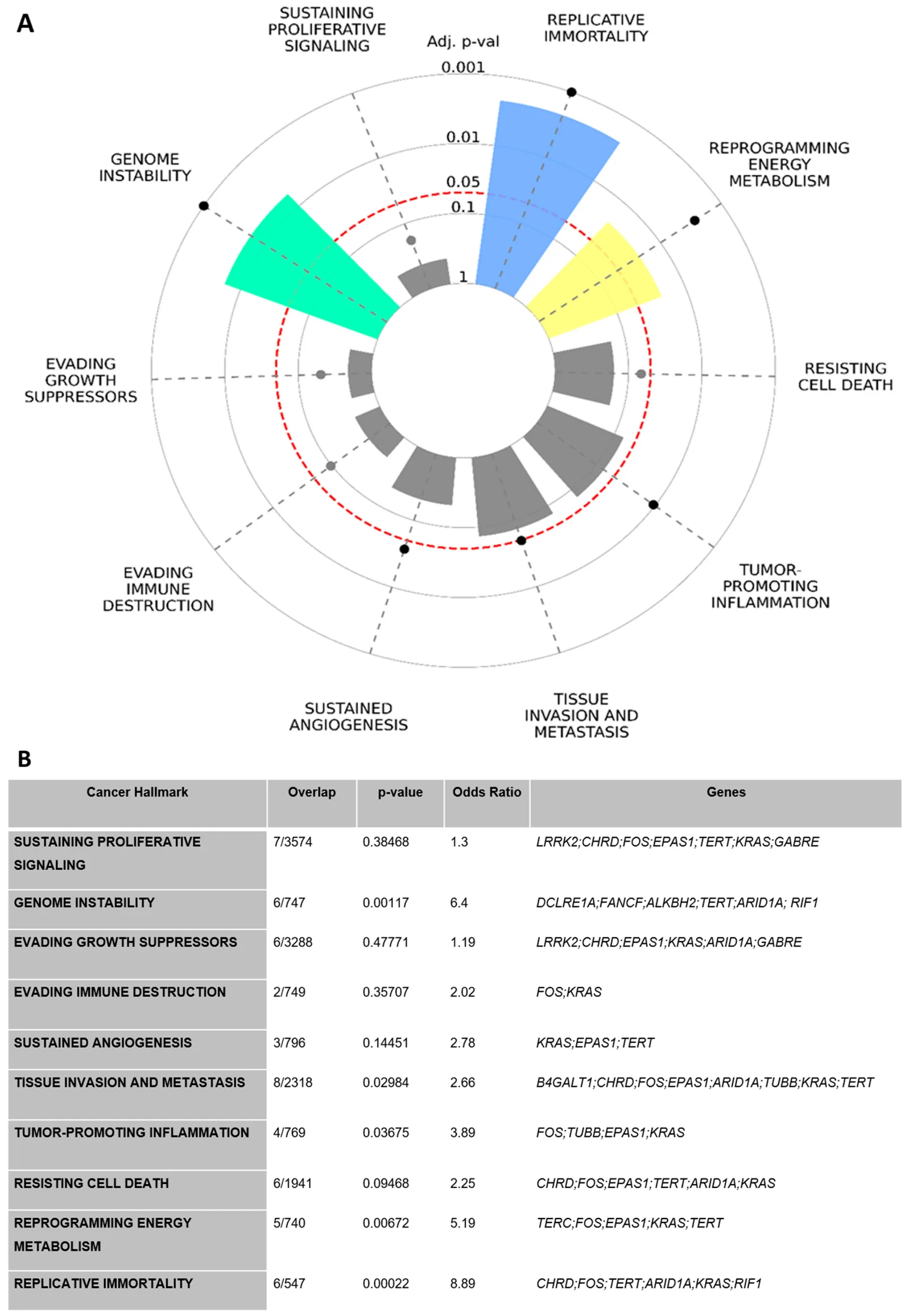
Enrichment analysis of cancer hallmarks for the analyzed gene set of top consensus biomarkers. Panel (**A**): Candidate genes were systematically curated and mapped onto established cancer hallmark categories, followed by enrichment and network-based analyses to elucidate dominant oncogenic processes. Radial bars indicate the strength of enrichment, expressed as adjusted *p*-values (outer rings denote higher statistical significance). Panel (**B**): Overview of cancer hallmarks and mapped consensus biomarker genes, reporting overlap counts, statistical significance, odds ratios, and contributing genes.

By comparing the enrichment of hallmarks in prognostic gene sets, distinct cancer hallmarks were identified in endometrioid ovarian cancer, forming a unique, tumor-specific cancer hallmark fingerprint. The circular enrichment plot (Figure 3A) summarizes the association between the analyzed gene set and the established hallmarks of cancer, with significance expressed as adjusted *p*-values. The top ten candidate genes were systematically curated and mapped onto established cancer hallmark categories, followed by enrichment and network-based analyses to elucidate dominant oncogenic processes. The analysis revealed a selective and non-random enrichment pattern, indicating that the investigated genes are functionally linked to specific biological processes rather than broadly distributed across all cancer hallmarks. A detailed overview is provided in Figure 3B, which summarizes the cancer hallmark enrichment analysis of consensus biomarker genes, reporting overlap counts, statistical significance, odds ratios, and contributions of the cancer hallmark fingerprint genes. A statistically significant enrichment is observed for genome instability (6/747 genes; *p* = 0.00117; OR = 6.4), replicative immortality (6/547 genes; *p* = 0.00022; OR = 8.89), and reprogramming energy metabolism (5/740 genes; *p* = 0.00672; OR = 5.19). These hallmarks are driven by genes central to DNA damage response and chromatin regulation, including *DCLRE1A*, *FANCF*, *TERT*, *ARID1A*, *RIF1*, and *ALKBH2*. The significant enrichment in the hallmark replicative immortality included the genes *CHRD*, *FOS*, *TERT*, *ARID1A*, *RIF1*, and *KRAS*, and reprogramming energy metabolism, mapped to the genes *TERC*, *FOS*, *EPAS1*, *KRAS*, and *TERT*.

### 3.4. Survival Analyses Stratified by LRRK2 Expression

**Figure 4:**
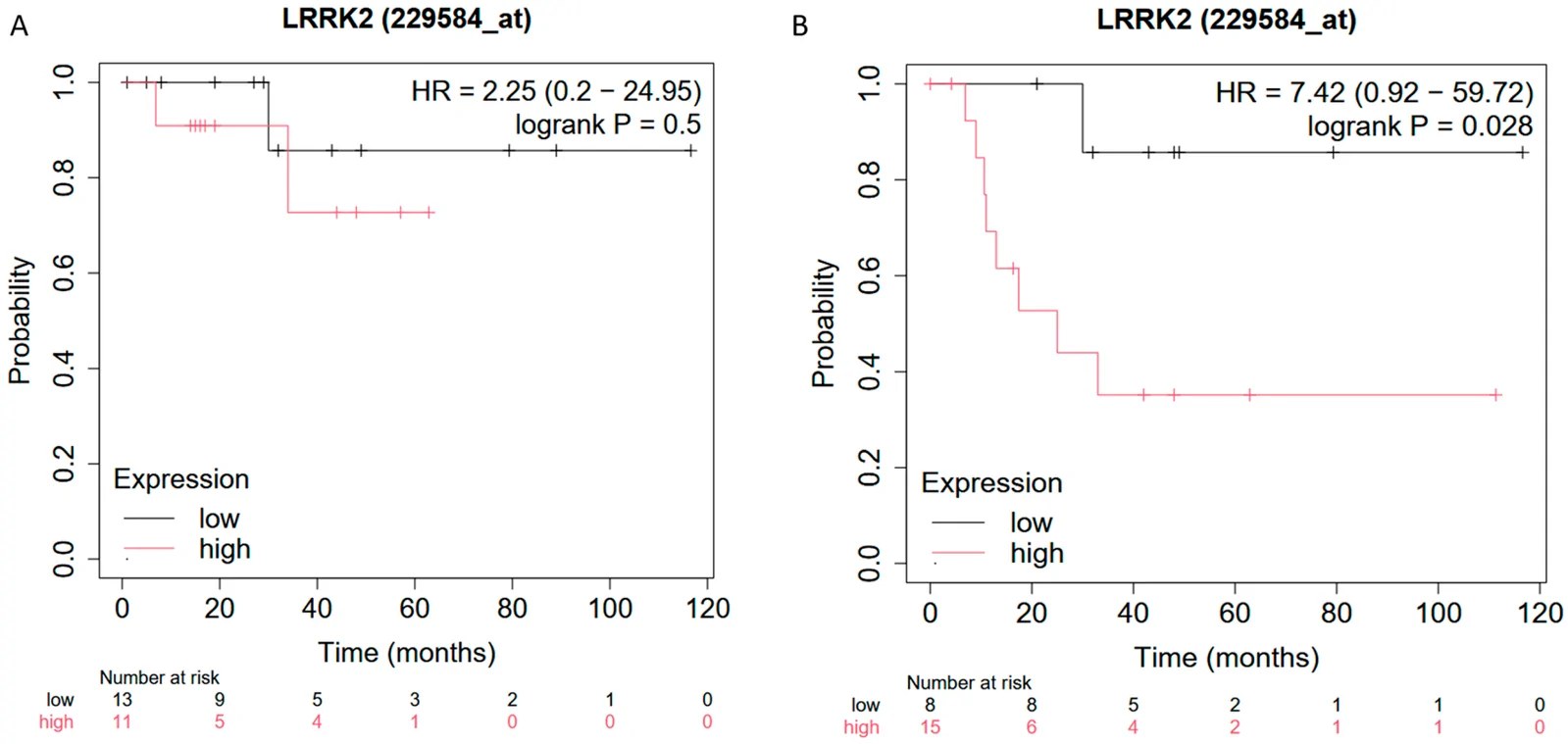
Stage-dependent prognostic impact of LRRK2 expression in endometrioid ovarian carcinoma. Here, Kaplan–Meier overall survival analyses stratified by LRRK2 expression levels (probe set 229584_at) in patients with endometrioid ovarian cancer are shown. Patients were dichotomized into high (red) and low (black) LRRK2 expression groups. Panel (**A**) depicts the survival probability in early-stage disease (FIGO stages I–II). Panel (**B**) depicts the results for advanced-stage disease (FIGO stages III–IV). Tick marks indicate censored observations. Numbers at risk at each time point are shown below the plots.

In patients with stage I–II tumors (Figure 4A), high LRRK2 expression was not significantly associated with overall survival. Although the hazard ratio suggested a trend toward increased risk in the high-expression group (HR = 2.25), the confidence interval was wide (95% CI: 0.2–24.95), and the difference between groups was not statistically significant (log-rank *p* = 0.5). Survival probabilities remained largely comparable between low and high expression groups over follow-up, indicating that *LRRK2* expression does not meaningfully stratify prognosis in early-stage disease. In contrast, among patients with stage III–IV endometrioid ovarian cancer, high *LRRK2* expression was associated with a markedly worse overall survival (Figure 4B). Patients with high *LRRK2* expression exhibited a steeper, earlier decline in survival probability than those with low expression. This difference reached statistical significance (log-rank *p* = 0.028), with a substantially elevated hazard ratio (HR = 7.42; 95% CI: 0.92–59.72). These findings imply that *LRRK2* may be linked to aggressive tumor behavior, therapy resistance, or disease progression, specifically in late-stage endometrioid ovarian cancer.

### 3.5. Identification of Mutations Influencing the Expression of KRAS and EPAS1 in Ovarian Endometrioid Ovarian Carcinoma

**Figure 5:**
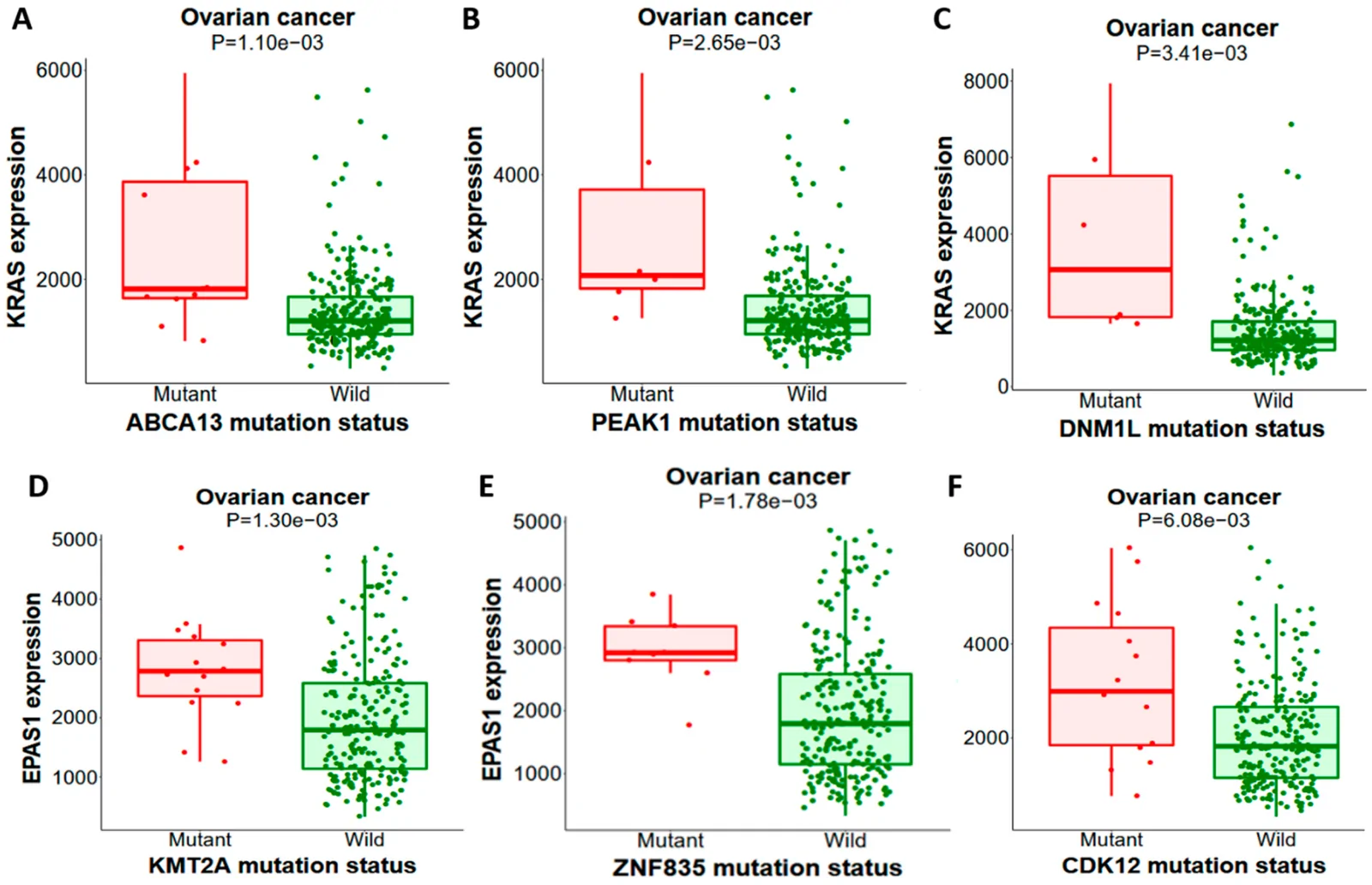
Association between selected gene mutations and *KRAS* and *EPAS1* expression in ovarian endometroid cancer. Panels (**A**–**C**) show *KRAS* mRNA expression stratified by *ABCA13* (**A**), *PEAK1* (**B**), and *DNM1L* (**C**) mutation status, respectively. Panels (**D**–**F**) depict EPAS1 mRNA expression stratified by *KMT2A* (**D**), *ZNF835* (**E**), and *CDK12* (**F**) mutation status, respectively. Boxplots display median, interquartile range, and full distribution of expression values. Statistical significance was determined using two-sided comparisons between mutant and wild-type groups; *p* values are indicated in each panel.

With the help of muTarget, which identifies statistical associations, we analyzed which gene mutations in ovarian endometrioid carcinoma influence the mRNA expression of *KRAS* and *EPAS1*. EC tumors with mutations of the genes *ABCA13* (Figure 5A; *p* = 1.10 × 10^−3^), *PEAK1* (Figure 5B; *p* = 2.65 × 10^−3^), and *DNM1L* (Figure 5C; *p* = 3.41 × 10^−3^) showed a clear increase in median mRNA expression levels of *KRAS* and a broader distribution, indicating enhanced *KRAS* pathway activation associated with these genomic alterations. Similarly, the *EPAS1* mRNA expression was significantly increased in tumors carrying mutations in the genes *KMT2A* (Figure 5D; *p* = 1.30 × 10^−3^), *ZNF835* (Figure 5E; *p* = 1.78 × 10^−3^), and *CDK12* (Figure 5F; *p* = 6.08 × 10^−3^). The findings suggest that mutations in *ABCA13*, *PEAK1*, and *DNM1L* may be associated with enhanced RAS/MAPK pathway activity, as reflected by elevated *KRAS* expression. Moreover, given the central role of *KRAS* in proliferation, survival, and metabolic reprogramming, these mutations may be associated with tumor aggressiveness through pathway amplification rather than direct *KRAS* mutation. Additionally, mutations in the genes *KMT2A*, *ZNF835*, and *CDK12* are associated with increased *EPAS1* expression, suggesting altered chromatin regulation and DNA damage response pathways in the activation of hypoxia-driven transcriptional programs. This may promote angiogenesis, metabolic adaptation, and treatment resistance. However, the identified associations for *KRAS* and *EPAS1* should be interpreted with caution, as they may reflect direct regulatory relationships, indirect effects, or co-occurrence with other molecular mutations and therefore should not be interpreted as evidence of causality. Finally, it is worth noting that the muTarget tool does not include a pre-screening for canonical activating of *EPAS* or *KRAS* hotspot mutations.

## 4. Discussion

Here, we present a novel, comprehensive, integrative transcriptomic, and machine learning-driven analysis that not only robustly distinguishes CC from EC but also uncovers novel consensus biomarkers and elucidates their functional landscape within the context of cancer hallmarks and aging. The sophistication of our approach, leveraging both LASSO and Random Forest algorithms, provides a powerful framework for identifying key molecular drivers that underpin the divergent pathologies of these ovarian cancer subtypes, a methodology increasingly recognized for its efficacy in uncovering prognostic and diagnostic biomarkers [45,46].

Our analysis identified 20 consensus biomarkers that robustly discriminate CC from EC samples, demonstrating substantial transcriptional reprogramming between these subtypes. Interestingly, *ALKBH2*, an m6A RNA demethylase, has been implicated in ovarian cancer prognosis, with related family members, such as *ALKBH5*, accelerating ovarian carcinogenesis through the NF-κB pathway [47,48,49]. The gene *DCLRE1A* is recognized for its critical roles in DNA replication, repair pathways (including the Fanconi anemia pathway and homologous recombination), and cell cycle regulation in epithelial ovarian cancer [50], underscoring its relevance to maintaining genomic integrity. The consistent subtype-specific expression of genes like *EPAS1* and *RPS28* further reinforces their potential as valuable diagnostic biomarkers, prompting further investigation into their functional roles [51]. Importantly, *RPS28* was included among the genes in this CC-associated ribosomal protein cluster, as shown in an integrated transcriptomic analysis of ovarian clear cell carcinoma [52]. The authors emphasized that dysregulated ribosomal proteins may participate in protein translation, proliferation, apoptosis, DNA-damage responses, and oncogenic signaling and suggested that ribosomal-protein genes may represent candidate tumor markers in CC [52].

Notably, the gene *EPAS1* is a key regulator of hypoxia signaling, oxygen homeostasis, and metabolic adaptation, and hypoxia pathways are tightly linked to cellular oxidative stress, stem cell maintenance, mitochondrial function, and lifespan regulation [53].

Beyond individual gene identification, the significant enrichment of pathways such as epithelial–mesenchymal transition, TNF-α signaling via NF-κB, glycolysis, and hypoxia is consistent with previous reports, which highlight their central roles in ovarian cancer progression and metastasis. Epithelial–mesenchymal transition (EMT) is among the enriched pathways, a critical process in cancer development, and its targeting is being explored to overcome chemoresistance in ovarian cancer [54,55]. The activation of hypoxia pathways, often regulated by genes like *EPAS1*, identified as an EC-upregulated gene in our study, is a well-established feature of clear cell and endometrioid ovarian carcinomas, reflecting adaptation to adverse tumor microenvironments [56,57]. Furthermore, the upregulation of *KRAS* signaling, a known oncogenic driver [58], coupled with the broad involvement of transcriptional regulatory mechanisms, indicates coordinated reprogramming of inflammatory, metabolic, and cell-state transitions in ovarian tumors. Interestingly, a recent mouse model for lung cancer revealed that aging represses the oncogenic *KRAS*-driven tumor initiation and growth [59].

A significant novel aspect of our study is the integration of our consensus biomarkers into a cancer hallmark-based functional landscape, explicitly linking them to aging processes. We observed a selective and non-random enrichment of specific cancer hallmarks: genome instability, replicative immortality, and reprogramming energy metabolism. These hallmarks are intimately tied to the aging process, reflecting molecular and cellular alterations that accumulate with age and predispose to malignant transformation [60]. The enrichment in genome instability, driven by genes central to DNA damage response and chromatin regulation, including *DCLRE1A*, *FANCF*, *TERT*, *ARID1A*, *RIF1*, and *ALKBH2*, underscores the importance of maintaining genomic integrity to prevent both aging-related decline and tumorigenesis. Notably, the gene *DCLRE1A*, also known as *SNM1A*, plays a role in DNA interstrand crosslink repair and response to genotoxic stress, and since DNA repair capacity declines with age, *DCLRE1A* is functionally relevant in aging tissues [61,62]. The gene *FANCF*, for example, is not only an ovarian cancer predisposing gene but also participates in DNA repair pathways crucial for genomic stability [63]. Similarly, the enrichment for replicative immortality, driven by genes such as *CHRD*, *FOS*, *TERT*, *ARID1A*, *RIF1*, and *KRAS*, highlights mechanisms that circumvent normal cellular senescence, a hallmark shared by both aging cells and cancer [64]. The gene *KRAS*, a member of the RAS GTPase family, is a proto-oncogene involved in cell proliferation, survival, and differentiation via the Raf/MEK/ERK signaling cascade [65,66]. Oncogenic *KRAS* mutations are found in approximately 14% of ovarian cancers, particularly in non-high-grade serous carcinoma subtypes and endometriosis-associated ovarian cancers (around 29%) [67,68]. In the context of ovarian aging, activating *KRAS* mutations have been shown to disrupt granulosa cell cycles, leading to deterioration of follicle growth. Moreover, aberrant *KRAS* activation in mouse theca-interstitial cells can result in female infertility [69]. These findings suggest that dysregulation of *KRAS* signaling can impact ovarian function and contribute to reproductive aging.

Finally, the enrichment in reprogramming energy metabolism, mapped to genes including *TERC*, *FOS*, *EPAS1*, *KRAS*, and *TERT*, reflects the metabolic rewiring observed in cancer cells to sustain rapid proliferation and survival, a process also influenced by aging-related metabolic dysregulation [70]. Our findings align with the broader understanding that ovarian cancer pathogenesis is influenced by metabolic reprogramming and cellular senescence, suggesting that targeting these age-related characteristics could offer novel personalized therapeutic strategies [71]. We propose that these aforementioned genes may represent components of, or molecular correlates of, a broader biological network involving hypoxia, oxidative stress, DNA damage responses, inflammation, and metabolic adaptation.

The survival analysis of *LRRK2* expression further highlights its stage-dependent prognostic significance in endometrioid ovarian cancer, representing a novel finding with potential clinical implications. It is worth noting that *LRRK2* is well known for its role in Parkinson’s disease and is linked to mitochondrial dysfunction, autophagy, and chronic inflammation, all of which are central aging pathways [72,73,74,75]. In our study, high *LRRK2* expression was significantly associated with a markedly worse overall survival in advanced-stage (III–IV) EC. This suggests that the *LRRK2* gene may not be merely a diagnostic marker but also a prognostic indicator, potentially linked to aggressive tumor behavior or therapy resistance, particularly in late-stage EC. Previous research has shown that *LRRK2* mutations in endometrial cancer are associated with a favorable prognosis [76] or that high *LRRK2* expression correlates with better prognosis in clear cell renal cell carcinoma [77]. Interestingly, contemporary research provided evidence that the inhibition of *LRRK2* accelerates the PARP inhibitor cytotoxicity via the inhibition of homologous recombination-mediated DNA double-strand break repair [78]. The latter-mentioned facts underscore the context-dependent nature of gene function in cancer, showing that the role of *LRRK2* can vary significantly across tumor types, specific genetic alterations (e.g., mutation vs. expression levels), and disease stage.

Interestingly, we found that mutations in *ABCA13*, *PEAK1*, and *DNM1L* appear to be associated with enhanced RAS/MAPK pathway activity, as evidenced by elevated *KRAS* expression. This suggests a mechanism where upstream genetic alterations can amplify *KRAS*-driven oncogenic processes, such as metabolic networks, which are critical for facilitating tumor survival and immune evasion across various cancer types [79,80]. Furthermore, our analysis revealed that mutations in *KMT2A*, *ZNF835*, and *CDK12* are associated with increased *EPAS1* expression. This implicates again altered chromatin regulation and DNA damage response pathways in the activation of hypoxia-driven transcriptional programs. Notably, hypoxia-inducible factors, particularly HIF-1α and HIF-2α (encoded by *EPAS1*), regulate the cellular response to low oxygen. These transcription factors induce genes involved in angiogenesis, metabolic reprogramming, and resistance to therapy [81]. HIF-2α was identified to be a critical regulator of chondrosarcoma progression, highlighting its role in governing malignancy-associated gene modules [82]. The earlier-mentioned mutation in *KMT2A*, a histone-lysine N-methyltransferase, suggests a role for epigenetic mechanisms in regulating *EPAS1* expression [83]. Similarly, the implication of *CDK12*, a gene known for its role in DNA damage response and transcriptional regulation, can impact the activation of HIF-2α-driven programs, representing a critical area for understanding how tumors adapt to their microenvironment and acquire aggressive traits, including resistance to conventional treatments. The identified associations for *KRAS* and *EPAS1* may reflect direct regulatory relationships, indirect effects, or co-occurrence with other molecular mutations and therefore should not be interpreted as evidence of causality.

## 5. Conclusions and Future Perspectives

In conclusion, our study leverages sophisticated integrative transcriptomic and machine learning analyses to provide a robust set of consensus biomarkers distinguishing ovarian clear cell and endometrioid carcinomas. This approach not only yields a highly accurate diagnostic classifier but also uncovers a nuanced functional landscape deeply intertwined with specific cancer hallmarks, hypoxia, and oxidative stress-related processes. Future functional studies are required to validate these biomarkers and explore their potential as therapeutic targets, ultimately improving clinical outcomes for patients with these challenging ovarian cancer subtypes. These functional studies incorporating pathway perturbation and experimental validation will be required to determine whether the identified mutations directly modulate *KRAS*-, *EPAS1*-, or other relevant signaling pathways, or whether the observed associations reflect indirect mechanisms or co-occurring genomic alterations.

## 6. Limitations

Several limitations should be considered when interpreting the present findings. First, the incomplete availability of clinical metadata, including nutritional status and previous treatment exposure, limits our ability to fully account for potential sources of residual confounding. Although these factors may influence transcriptomic profiles, the present study was primarily designed as an exploratory investigation of molecular differences between ovarian cancer subtypes. The absence of complete metadata for all potentially relevant clinical variables in the publicly available discovery cohort does not eliminate the scientific value of the present analysis. On the contrary, we consider the identification of these candidate biomarkers to represent an important starting point for subsequent translational and mechanistic research. The reported diagnostic performance, including the cross-validated AUC, should be interpreted as an internally validated estimate rather than definitive evidence of clinical utility. Second, the genes identified in this study should be considered candidate transcriptomic biomarkers rather than clinically validated diagnostic biomarkers. The translational relevance of candidates such as *RPS28*, *EPAS1*, *ALKBH2*, *DCLRE1A*, and *LRRK2* will require prospective studies in independent, clinically well-characterized patient cohorts, together with orthogonal validation at both the RNA and protein levels. In particular, immunohistochemical and immunofluorescence validation in clinical tumor specimens, complemented where feasible by studies in cellular and organoid models, will be important to establish the reproducibility, biological relevance, and diagnostic potential of these candidates. Such investigations represent an essential next step before these markers can be considered for clinical diagnostic implementation. Similarly, the mutation–expression associations identified using the muTarget approach should be interpreted as statistical associations rather than evidence of direct causal regulation.

## Data Availability

Gene expression data were obtained from the NCBI Gene Expression Omnibus (GEO) dataset GSE73614, profiled on the Agilent-014850 Whole Human Genome Microarray 4x44K platform (GPL6480).

## Abbreviations

The following abbreviations are used in this manuscript:

CC	Clear cell carcinoma
EAOC	Endometriosis-associated ovarian cancer
EC	Endometroid carcinoma
